# Sphingomonas Paucimobilis Pneumonia Complicated by Empyema in an Immunocompetent Patient: A Case Report and Concise Review of Literature

**DOI:** 10.7759/cureus.24820

**Published:** 2022-05-08

**Authors:** Neil R Kumar, Bridget S Norwood

**Affiliations:** 1 Internal Medicine, Jackson Memorial Hospital, University of Miami, Miami, USA; 2 Internal Medicine, Miller School of Medicine, University of Miami, Miami, USA

**Keywords:** diabetes mellitus, aspiration, pleural effusion, immunocompetent, empyema, pneumonia, sphingomonas paucimobilis

## Abstract

*Sphingomonas paucimobilis* is an aerobic, Gram-negative bacterium that is found widely in the environment and on hospital equipment. Although this organism usually causes infection in immunocompromised patients, it may cause pulmonary disease in immunocompetent patients, in rare cases. We report a case of *Sphingomonas paucimobilis* pneumonia complicated by empyema in an immunocompetent patient. We present a case of a 59-year-old female who was admitted for a congestive heart failure exacerbation and pneumonia. After imaging confirmed pneumonia and pleural effusion, monotherapy with levofloxacin was started. Thoracentesis revealed empyema caused by *Sphingomonas paucimobilis*. Despite chest tube placement, thoracoscopy with decortication was required due to continued clinical deterioration. After surgical intervention and an adjusted antibiotic regimen of cefepime, the patient clinically improved and was discharged. Upon follow-up, she had recovered completely with no residual disease. Alongside a concise review of the literature, our study highlights the importance of this infection in immunocompetent patients and advises providers to identify causes of aspiration when *Sphingomonas paucimobilis* empyema is diagnosed.

## Introduction

*Sphingomonas paucimobilis*, formerly known as *Pseudomonas paucimobilis*, is an aerobic, non-fermenting Gram-negative bacterium that can survive in low nutrient environments [1]. It has been commonly isolated from the natural environment, especially from water and soil and hospital sources including the hospital water system and laboratory instruments [2]. It has been found in a wide variety of clinical specimens including blood, sputum, urine, and cerebrospinal fluid. Analyzing *Sphingomonas paucimobilis* cases throughout the world over a span of 30 years, a retrospective study concluded that the highest percentage of infections caused by *Sphingomonas paucimobilis* was bacteremia/septicemia (38%), followed by peritonitis (10%). This study documented pneumonia/lung infections at 6%, with only 2% of *Sphingomonas paucimobilis* cases with empyema [1]. With only three previously reported cases of empyema caused by this organism, we present a case of a 59-year-old immunocompetent female who developed *Sphingomonas paucimobilis *pneumonia complicated by empyema.

This article was previously presented as a poster at the University of Miami, Department of Medicine Eighth Annual Eugene J. Sayfie, MD Research Day on March 16, 2022.

## Case presentation

A 59-year-old female presented with gradual but progressively worsening dyspnea of two weeks' duration. This was associated with worsening peripheral edema and a four-day history of productive cough with yellow sputum. However, she denied fevers, chills, or chest pain. Her medical history revealed congestive heart failure with a reduced ejection fraction of 10-20%, well-controlled diabetes mellitus with recent glycated hemoglobin (HbA1c) of 5.7%, hyperlipidemia, and hypertension. Her home medications included metformin, furosemide, valsartan, and carvedilol. She worked as a school crossing guard. On examination, she had normal vitals except tachypnea of 26. Her oxygen saturation was 93% on room air and she had expiratory wheeze in the right lung base with mild crackles.

**Figure 1:**
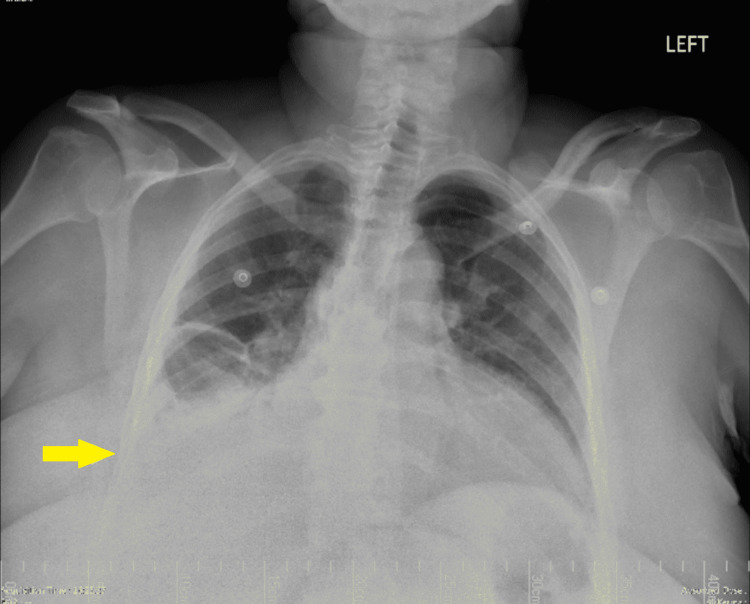
Chest x-ray showing a right basilar consolidation and effusion (yellow arrow).

**Figure 2:**
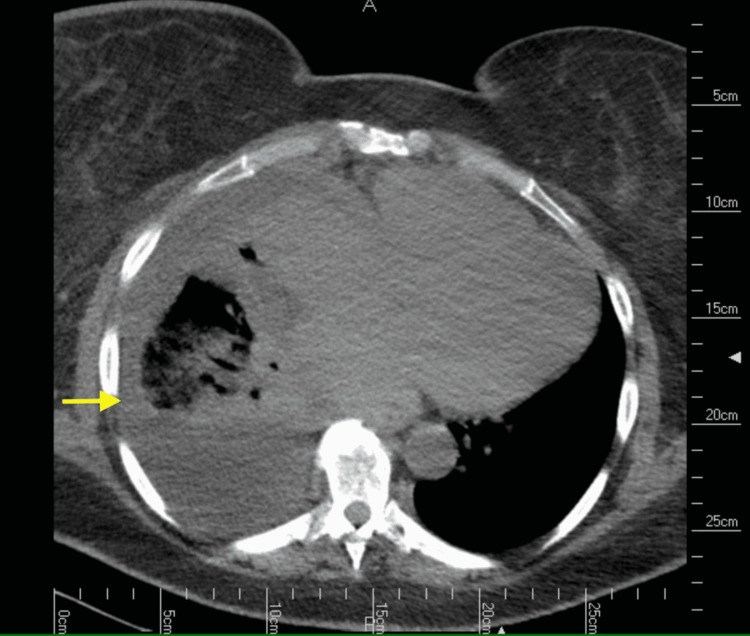
Chest CT scan (axial view) showing a right lower lobe pneumonic consolidation with a moderate-sized pleural effusion (yellow arrow).

Laboratory studies on admission showed a white blood cell count of 11.42 x 10^3^/uL with a left shift noted as neutrophilia at 86.6%, a brain natriuretic peptide level of 2039, normal electrolytes, and negative troponins. Electrocardiogram displayed no signs of cardiac ischemia. A chest x-ray showed a right basilar consolidation and effusion (Figure 1). Follow-up chest computed tomography (CT) scan revealed a right lower lobe pneumonic consolidation with a moderate-sized pleural effusion (Figure 2).

**Figure 3:**
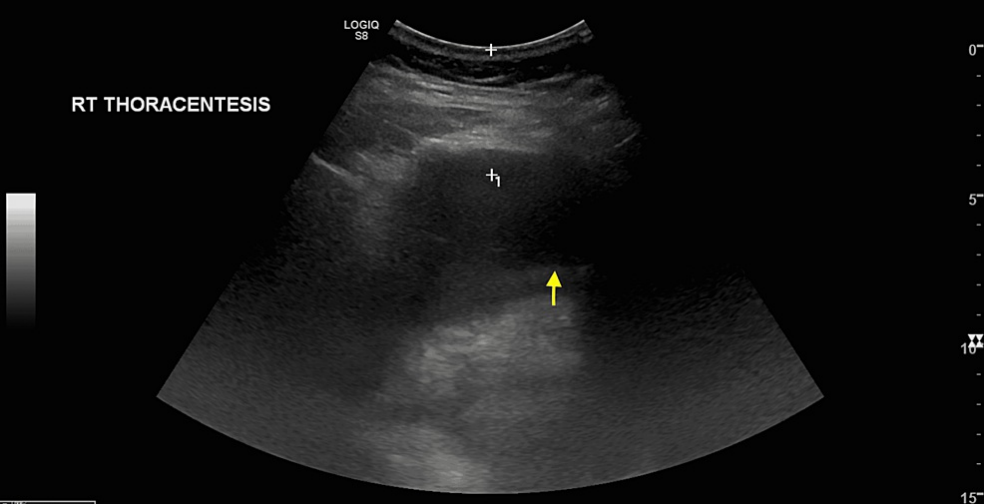
Ultrasound showing a moderate-sized pleural fluid pocket (yellow arrow).

The presenting dyspnea was evaluated to be likely multifactorial, secondary to acute on chronic systolic congestive heart failure exacerbation, and right-sided pleural effusion with lower lobe pneumonia. Treatment with intravenous levofloxacin 750 mg daily was initiated. Ultrasound confirmed a moderate-sized pleural fluid pocket despite diuresis and thoracentesis was completed, draining 925 cc of cloudy yellow fluid (Figure 3). Pleural fluid culture revealed one organism: *Sphingomonas paucimobilis*.

**Figure 4:**
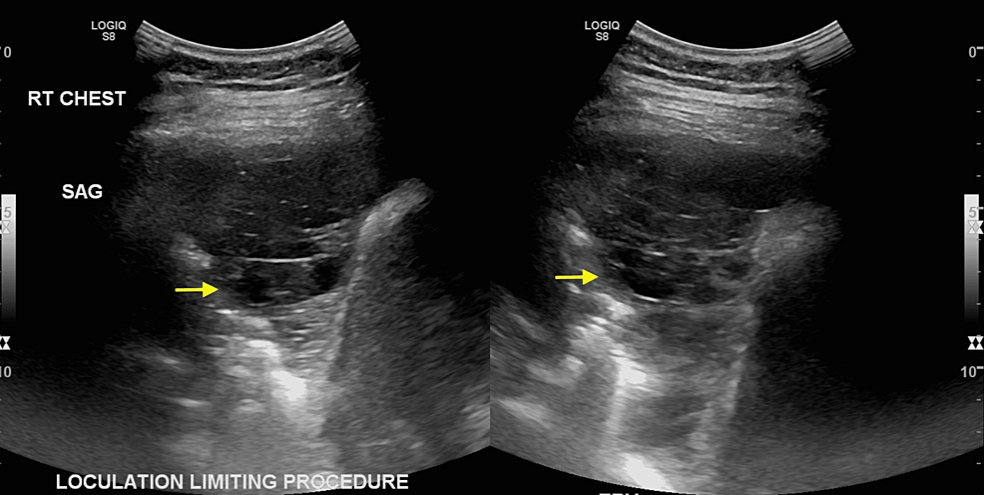
Ultrasound showing a multiloculated right-sided pleural effusion (yellow arrows).

Culture data revealed sensitivity to ceftriaxone and cefepime with a minimum inhibitory concentration (MIC) dilution of ≤1, sensivitity to piperacillin/tazobactam with a MIC dilution of ≤4, and intermediate susceptibility to levofloxacin with a MIC dilution of 4. She was initially treated with an antimicrobial regimen of intravenous ceftriaxone 2 g daily and chest tube placement. Despite a one-week course of antibiotics and drainage of the empyema, the patient continued to have dyspnea with diminished breath sounds on the right side and leukocytosis of 14.53 x 10^3^/uL. Per infectious disease recommendations, antibiotics were escalated to 2 g every eight hours of intravenous cefepime. Repeat ultrasound revealed a multiloculated right-sided pleural effusion (Figure 4).

**Figure 5:**
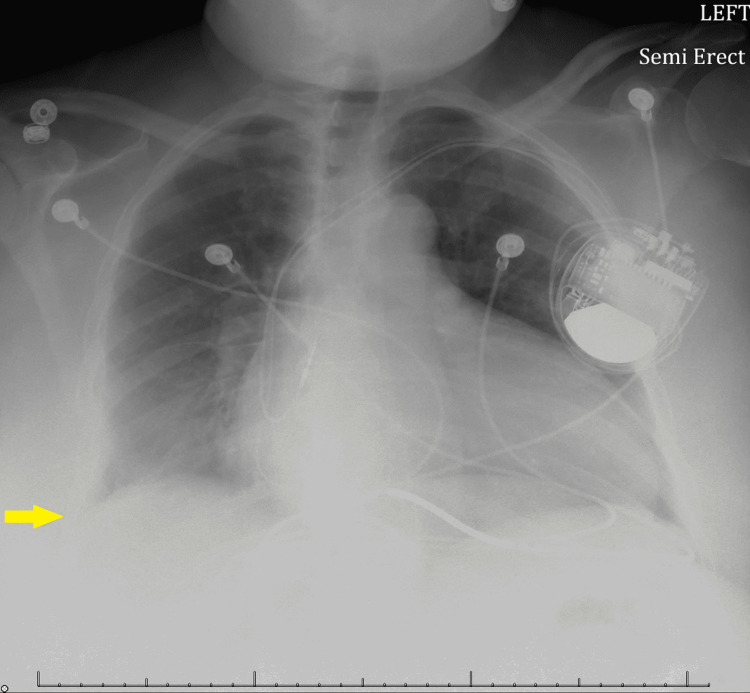
Chest x-ray showing complete resolution of the previous consolidation and pleural effusion on the right side (yellow arrow). Between hospitalizations, an automatic implantable cardioverter defibrillator was inserted for primary prevention of sudden cardiac death in the setting of the patient's severely reduced ejection fraction and New York Heart Association class II functional status.

She underwent a right thoracoscopy converted to a right thoracotomy and right lung decortication. Her symptoms of dyspnea and cough, leukocytosis, and oxygen saturation slowly improved. She was then discharged to subacute rehabilitation with a regimen of intravenous cefepime 2 g every eight hours to complete a two-week course. Even though she was subsequently admitted to the same institution with a congestive heart failure exacerbation, a follow-up chest x-ray revealed complete resolution of the previous consolidation and pleural effusion on the right side (Figure 5).

## Discussion

*Sphingomonas paucimobilis* empyema is a rare condition with only three previously reported cases. This organism causes a variety of community-acquired and nosocomial infections, normally occurring in immunocompromised hosts [3]. In Taiwan, a retrospective study examined 55 cases of *Sphingomonas paucimobilis* infections over a span of five years examining relevant data including underlying diseases. Diabetes mellitus was the most prominent risk factor for *Sphingomonas paucimobilis* infections, followed by malignancy, chronic heart disease, and alcoholism [2]. Another study, also from Taiwan, concluded that the most common comorbidities identified in cases of *Sphingomonas paucimobilis* bacteremia were malignancy, immunosuppressant use, and diabetes mellitus [4].

All three other previously reported cases of *Sphingomonas paucimobilis* empyema had at least one of these risk factors as well as a similarity in the location and origin of the infection. The first case by Cover et al., in 1988 in Pennsylvania, USA, described a 56-year-old man who had just received orthotopic cardiac transplantation. This patient had been on an immunosuppressive regimen of cyclosporine, prednisone, and azathioprine for several weeks before developing a right lower lobe pleural effusion infected by *Sphingomonas​​​​​​​ paucimobilis* and associated oral flora. This study went on to conjecture that *Sphingomonas​​​​​​​​​​​​​​ paucimobilis *was part of that patient’s oral flora, leading to empyema secondary to aspiration pneumonia [5]. Another case in Virginia, USA, demonstrated that a 36-year-old man with a past medical history significant for alcohol dependence developed *Sphingomonas​​​​​​​​​​​​​​ paucimobilis* community-acquired pneumonia with bilateral lower lobe empyemas [6]. Alcoholism predisposes patients to the aspiration of bacteria, often that of indigenous oral flora, which can lead to aspiration pneumonia [7,8]. The last reported case in the literature occurred in 2018 in Massachusetts, USA, when a 77-year-old man developed *Sphingomonas​​​​​​​​​​​​​​ paucimobilis* empyema secondary to foreign body aspiration. Imaging confirmed a metallic foreign body from a remote dental procedure within the right lower lobe bronchus of this patient, leading to the rare complication of empyema. This individual’s main risk factor was non-insulin-dependent diabetes mellitus; however, he was well controlled with an HbA1c of 6.7% [9].

This most recent case of *Sphingomonas​​​​​​​​​​​​​​ paucimobilis* empyema draws a parallel to our case in the sense that both immunocompetent patients had the most significant risk factor for *Sphingomonas​​​​​​​​​​​​​​ paucimobilis* infections, diabetes mellitus; however, in both cases, the patients were well controlled with an HbA1c below 7%. This point illustrates that while most *Sphingomonas​​​​​​​​​​​​​​ paucimobilis* infections are associated with immunocompromised patients, well-controlled diabetes mellitus may increase the risk of severe *Sphingomonas​​​​​​​ paucimobilis* infections, particularly empyema, in patients without blatant immunosuppression [3]. In fact, a recent primary care cohort study in 2018 showed evidence that compared to patients without diabetes mellitus, those with diabetes mellitus and optimal control (HbA1c between 6% and 7%) have elevated risk of hospitalization and infection [10]. There was also a positive correlation between rising HbA1c (most evident with HbA1c ≥11%) and increased rates of a wide range of infections including bone/tissue infection, endocarditis, and pneumonia [10]. This may be due to the level of immune dysfunction that occurs in even well-controlled diabetic patients.

A literature review revealed another correlation to our case: all four cases of empyema occurred in the right lower lobe of the lung, with one case also occurring bilaterally. In ambulatory patients, aspiration pneumonia occurs in the dependent pulmonary segments which are classically the lower lobes of the lung, especially the right [11,12]. In fact, the cause of all three previously reported cases was traced or postulated to be due to direct aspiration or oral bacteria translocation. Unfortunately, the source of the *Sphingomonas​​​​​​​​​​​​​​ paucimobilis* in our case was not determined; however, the location of our patient’s empyema along with the other three cases indicates that the risk of *Sphingomonas​​​​​​​​​​​​​​ paucimobilis* empyema may be increased in patients with risk factors for oral bacteria translocation including advanced age, alcoholism, impaired airway clearance, and poor dental hygiene.

## Conclusions

*Sphingomonas paucimobilis* is an organism that has caused a wide variety of both nosocomial and community-acquired infections. We report a rare cause of *Sphingomonas paucimobilis *pneumonia complicated by empyema in an immunocompetent patient. Despite a severe infection, *Sphingomonas paucimobilis* empyema responds to standard treatment of decortication with complete recovery. While *Sphingomonas paucimobilis* infections are usually seen in immunocompromised patients, our case suggests that controlled diabetes mellitus must be examined as a risk factor for *Sphingomonas​​​​​​​ paucimobilis* empyema. Furthermore, we identified that all documented *Sphingomonas​​​​​​​ paucimobilis *empyemas have occurred in the right lower lobe of the lung, drawing a possible association of *Sphingomonas​​​​​​​ paucimobilis* to oral bacteria translocation. Thus, we advise physicians to investigate causes of aspiration in patients with *Sphingomonas paucimobilis* empyema.
